# NRBF2 plays a crucial role in the acquisition process of learning and memory, independent of the Vps34 complex

**DOI:** 10.3389/fnbeh.2025.1529522

**Published:** 2025-02-12

**Authors:** Songfen Wu, Haicai Zhuang, Xidan Zhou, Kuan Li

**Affiliations:** ^1^School of Pharmacy, Guangdong Medical University, Zhanjiang, China; ^2^Department of Pharmacy, Shenzhen People’s Hospital, The Second Clinical Medical College, Jinan University, Guangzhou, China; ^3^The First Affiliated Hospital, Southern University of Science and Technology, Shenzhen, China; ^4^The Brain Cognition and Brain Disease Institute, Shenzhen Institutes of Advanced Technology, Chinese Academy of Sciences, Shenzhen, China

**Keywords:** Nrbf2, VPS34, autophagy, memory acquisition, LTP

## Abstract

**Introduction:**

NRBF2, a component of autophagy-associated PIK3C3/VPS34-containing phosphatidylinositol 3-kinase complex, plays a crucial role in learning and memory processes, yet its specific impact on memory and the underlying molecular mechanisms remains unclear.

**Methods:**

Here, we utilized NRBF2 knockout mice to examine its influence on the time course of fear memory. Employing quantitative PCR, Western blot analysis, behavioral tests, and electrophysiology, we investigated the mechanisms through which NRBF2 affects memory processing.

**Results:**

We observed an increase in *Nrbf2* mRNA levels at 6 and 12 h, and protein levels at 6 h post fear conditioning. Depletion of NRBF2 impaired memory acquisition, short-term, and long-term memory without causing any anxiety-like behavior. Interestingly, inhibition of Vps34 and autophagy by SAR405 disrupted fear memory consolidation, while leaving memory acquisition, short-term memory, and long-term potentiation (LTP) unaffected. Our results suggested that NRBF2 deletion impaired memory acquisition through an autophagy-independent pathway and provided novel insights into the role of NRBF2 in the central nervous system.

**Discussion:**

This study offer new insights into the role of NRBF2 and highlight the potential of targeting NRBF2 as a therapeutic strategy for addressing cognitive deficits associated with various disorders.

## 1 Introduction

Autophagy is an evolutionarily conserved catabolic process that encompasses the initiation, nucleation, elongation, maturation, fusion, and degradation stages (Li et al., 2020). This process plays a pivotal role in neurodevelopment and neurotransmitter release (Kuijpers and Haucke, 2021), contributing to the maintenance of neuronal integrity and synaptic plasticity (Nikoletopoulou et al., 2017). Impairments in autophagy are associated with memory deficits in aging and Alzheimer’s disease (AD) (He and Klionsky, 2009; Nixon, 2013; Yang et al., 2017). Nuclear Receptor Binding Factor 2 (NRBF2) is initially identified as a coregulator that interacts with nuclear receptors to modulate transcriptional activity (Yasumo et al., 2000; Flores et al., 2004). Recent studies have revealed NRBF2 as the fifth subunit of the active PIK3C3/VPS34-containing class III phosphatidylinositol 3-kinase (PtdIns3K) complex, a critical component of autophagy (Young et al., 2016; Ohashi, 2021). Accumulating evidence has suggested that NRBF2 is crucial for autophagosome formation and maturation. Through its MIT domain, NRBF2 directly interacts with Atg14L, enhancing Vps34 kinase activity and facilitating autophagy initiation (Lu et al., 2014). Moreover, NRBF2 acts as a RAB7 effector, supporting autophagosome maturation (Cai et al., 2021). Both NRBF2 and autophagy have been implicated in the regulation of learning and memory processes (Lachance et al., 2019; Hylin et al., 2018; Li et al., 2019).

Unique molecular mechanisms are known to underlie different stages of memory formation, specifically the acquisition, consolidation, and reconsolidation (Johansen et al., 2011). Learning and acquisition are the basis of memory, while consolidation is a process of the conversion of labile short-term memory (STM) into stable long-term memory (LTM) (McGaugh, 2000; Nader and Hardt, 2009; Bush et al., 2010). Synaptic plasticity is fundamental to learning and memory, and the abnormality of its two different forms, long-term potentiation (LTP) and long-term depression (LTD), is regarded as cellular mechanisms underlying memory deficits (Bin Ibrahim et al., 2022). Our previous study found that inhibiting autophagy in basolateral amygdala, either by targeting the Vps34 or Atg5, resulted in increased inhibitory synaptic transmission and impaired LTM, while preserving memory acquisition and STM integrity (Li et al., 2019). Two other studies have also demonstrated that autophagy inhibition is essential for LTD induction (Shehata et al., 2012; Pan et al., 2021). The involvement of NRBF2 in memory has also been previously documented. *Nrbf2* knockout (NRBF2-KO) mice exhibited memory deficits and decreased autophagy in hippocampus, accompanied by decreased LTP (Lachance et al., 2019), whereas *Nrbf2* conditional knockout mice in the nervous system showed impaired spatial memory with minimal autophagy deficits, indicating an autophagy-independent pathway (Ouyang et al., 2020). These findings have led to the intriguing hypothesis that NRBF2 may influence memory formation and LTP through non-autophagy pathways. However, the precise role of NRBF2 in each memory stage and its association with autophagy in memory regulation remains to be fully elucidated.

To investigate whether NRBF2 affects the acquisition and consolidation of fear memories, particularly through the autophagy pathway, we employed fear conditioning tests and field potential recording techniques to explore the involvement of NRBF2 in the memory process. Additionally, SAR405, a Vps34 inhibitor, was used to elucidate the underlying association with autophagy. Our results showed that depletion of NRBF2 impaired memory acquisition and subsequent STM and LTM, while inhibition of autophagy by SAR405 disrupted fear memory consolidation, without altering memory acquisition, short-term memory, and LTP. These results suggest that NRBF2 deletion impairs memory acquisition through an autophagy-independent pathway and provide novel insights into the role of NRBF2 in the central nervous system.

## 2 Materials and methods

### 2.1 Animals

*NRBF2*^–/–^ mice utilized in this study were acquired from Prof. Jia-Hong Lu (University of Macau, Taipa, Macau SAR China). NRBF2 heterozygous mutant mice were bred with C57BL/6 J mice and interbred to generate the *NRBF2*^–/–^ mice. All animals were housed under a 12/12 h light/dark cycle with lights on at 7:00 AM, and maintained at a constant temperature (22 ± 2 °C) and humidity of 50 ± 10%, with *ad libitum* access to food and water. Five mice were acclimated per cage, and male mice aged 8–11 weeks were used for the experiment. All experimental procedures were approved by the Animal Welfare Committee of Huazhong University of Science and Technology and the Ethics Committee at the Shenzhen People’s Hospital, in accordance with the ARRIVE guidelines 2.0 (Percie du Sert et al., 2020). All methods were carried out following with the relevant guidelines and regulations. At the end of the experiments, the mice used for behavioral testing were euthanized in a CO_2_ chamber.

### 2.2 Quantitative PCR (qPCR)

Total RNA was extracted from hippocampal homogenates of 8–10-week-old wild-type littermate mice using TRIzol^®^ Reagent (Invitrogen, Cat# 15596018CN) following the manufacturer’s protocol. A quantity of 1 μg of RNA was then reverse transcription to generate cDNA libraries was performed using the cDNA synthesis kit (Vazyme, Cat# R212-02) according to the provided protocol. Quantitative PCR (qPCR) was conducted with the Universal SYBR qPCR Master Mix (Vazyme, Cat# Q511-02). Gene expression analysis was carried out using the 2^–Δ^ ^Δ^ *^CT^* method. The primers used for transcript analysis were as follows: Nrbf2 - F: AAG GAC CCC TCA ACC TTG CT, Nrbf2 - R: CAG TTC CAG TGA TAA GTG AGC C; GAPDH - F: AAC GAC CCC TTC ATT GAC, GAPDH - R: TCC ACG ACA TAC TCA GCA C.

### 2.3 Western blotting (WB)

The procedures were processed according to our previous protocol with minor modifications (Li et al., 2019; Shi et al., 2018). In brief, hippocampal tissue from each mouse was homogenized in RIPA buffer (MCE, HY-K1001), supplemented with 1 mM PMSF, 1 × protease inhibitor cocktail and 1 × phosphatase inhibitor cocktail (Sigma). Samples were centrifuged at 12,000 g for 20 min at 4°C. Lysates were heated at 100°C with 6× loading buffer (Beyotime) for 10 min and then stored at −20°C until next step. A total of 20 μg protein per sample was loaded on 10% SDS-PAGE gel, and then transferred onto PVDF membranes (Millipore) and blocked in 5% bovine serum albumin (BSA) in Tris-buffered saline containing 0.1% Tween-20 (TBST) for 2 h at room temperature. The transferred membranes were incubated overnight at 4°C with primary antibodies: NRBF2 rabbit mAb (1:1000, Proteintech, 24858-1-AP) and GAPDH mouse mAb (1:60000, Invitrogen, AM4300). After three washes with TBST, the membranes were then incubated with horseradish peroxidase (HRP)-conjugated secondary antibodies (1:2000) in TBST with 1% BSA for 2 h at room temperature. Following additional washes, the membranes were reacted with an enhanced chemiluminescence reagent (Epizyme). Images were scanned and captured with Micro Chemi (Bio-rad, ChemiDoc XRS+) and the optical densities of the detected bands were quantified using ImageJ software (NIH).

### 2.4 Open-field test

The open field test (OFT) is commonly used to assess spontaneous locomotor activity and exploratory behavior in response to novel environments in rodents (Sun et al., 2024). The OFT was conducted as described in a previous study with minor modifications (Shi et al., 2018; Shoji and Miyakawa, 2021). Before the experiment, the mice were habituated to the investigator’s handling for 180 s on three consecutive days in the laboratory where the mice were subjected to the OFT. The mice were placed in a square open arena (40 cm × 40 cm × 40 cm), and allowed to explore the area freely for 20 min. Their activity was recorded and analyzed using the Tru Scan Activity System (Coulbourn Instruments). The central area was illuminated to 100 lx using LED lights mounted on the ceiling of each apparatus. The arena surface was cleaned with 70% ethanol after each trial.

### 2.5 Elevated-plus maze

The elevated plus maze (EPM) apparatus consists of a central area (5 cm × 5 cm), two open arms (25 cm × 5 cm), and two enclosed arms (25 cm × 5 cm × 15 cm). The maze is positioned 50 cm above the ground in a room. The EPM procedure was performed as previously described in a study, with minor modifications (Painsipp et al., 2008). The light intensity at the central quadrangle was 70 lux, on the open arms 80 lux and in the closed arms 40 lux. The mice were placed in the central square, facing one of the open arms, and were allowed 5 min to explore the maze. The time spent by each mouse in each arm and the number of arm entries during the 5-min exploration were recorded using the DigBehv Animal Behavior Analysis System.

### 2.6 Light-dark box

The light-dark (LD) box apparatus (45 cm × 27 cm × 27 cm) consisted of two compartments: a light compartment and a dark compartment, separated by an opaque plexiglass partition with a 5 cm diameter hole. This hole allowed the mouse to access the surrounding arena. The LD test was performed following our previously established protocol, with minor adjustments (Shi et al., 2018). The light compartment was illuminated with a strong light source (400 lux). The mice were individually placed in the center of the light compartment, facing away from the hole, and were allowed to explore the apparatus freely for 10 min. The time spent by the mice in the light and dark compartments was recorded using the DigBehv Animal Behavior Analysis System.

### 2.7 Fear conditioning

The fear conditioning test was performed according to our previous protocol with minor adjustments (Li et al., 2019; Wilensky et al., 2006). All the experiments were conducted in two contexts: context A and context B. The conditioning chamber (context A, 32 cm × 26 cm × 30 cm) was sound-attenuating, with foot shocks administered through a stainless grid floor. The test chamber (context B, 32 cm × 26 cm × 30 cm) was brightly illuminated and featured a flat black plastic floor. The apparatus was illuminated by a light source with an intensity of 20 lux. The chambers were cleaned with 70% ethanol before each session. Before the experiment, the mice were habituated to the investigator’s handling for 180s on three consecutive days in the laboratory, where they would later undergo the fear conditioning test. On day 1 (habituation), the mice were introduced to context A and allowed to freely explore for 5 min. On the training day (day 2), a tone conditioned stimulus (CS, 80 dB) was presented for 29 s that co-terminated with a single electric foot shock as an unconditioned stimulus (US, 0.7 mA, 1 s). This pairing was repeated five times, with a 60-s interval between CS-US presentations. The mice were returned to their home cages 30 s after the last foot shock. Fear memory test was performed at 3 h and 24 h after fear conditioning. During the memory test in context B, the mice were exposed to five tones (30 s each) with a 30-s interval. Fear memory was assessed by quantifying the percentage of freezing time during the test periods.

For SAR405 treatment, SAR405 (1 μM, ApexBio) was intracranial injected to the hippocampus. The mice were then subjected to the habituation procedure in conditioning chamber, and fear conditioning training was performed 24 hours after the injection, as described above.

### 2.8 Intra-hippocampus microinjections

C57BL/6 J mice were anesthetized with pentobarbital sodium (60 mg/kg, i.p.) and then mounted in a stereotaxic apparatus (RWD Life Science, China). 22-gauge stainless steel guide cannulas were bilaterally implanted dorsal to the CA1 region (AP: −1.7, ML: ± 1.5, DV: −1.8). They were then given 1 week to recover from surgery. When intracranial injection was performed before fear conditioning, a 33-gauge injection cannula was used to replace the inner sealing wire and protruded 1 mm beyond the guide cannula. Drugs were infused into the hippocampus at a rate of 0.5 μL/min with a total volume of 1.0 μL/side. SAR405 (1 μM, ApexBio) was used in the intra-hippocampus injections.

### 2.9 Electrophysiological recording

Field excitatory postsynaptic potentials (fEPSPs) were recorded using the field potential recording technique, following our previous protocol with slight modifications (Wang et al., 2015; Wu et al., 2022). In brief, the brains of C57BL/6J mice, which did not undergo any behavioral experiments, were sectioned into 400 μm-thick coronal slices containing the hippocampus using a microslicer (Leica VT1000 S; Leica Biosystems, Germany). The slices were incubated in artificial cerebrospinal fluid (ACSF) for at least 1.5 h at 27 °C. Subsequently, individual slice was transferred to a perfusion-type recording chamber and continuously superfused with ACSF pre-gassed with 95% O2/5% CO2 using a constant-current pump (HL-2, Shanghai JingDa Biochemical Instrument). A bipolar electrode was positioned in the Schaffer collaterals, and fEPSPs were recorded in the CA1 stratum radiatum layer using a glass micropipette filled with 3 M NaCl (2–5 MΩ, pulled from borosilicate capillaries). The recordings were collected and analyzed using a multi-channel physiological signal acquisition and processing system (RM6240BD, Chengyi, China). The stimulation intensity was gradually increased, and the corresponding fEPSP amplitudes were recorded. The stimulation intensity was then adjusted to produce a basal fEPSP amplitude corresponding to 1/3 to 1/2 of the maximum fEPSP amplitude, with a stimulation frequency of 0.033 Hz and an inter-stimulus interval of 30 s. After recording stable fEPSPs for at least 15 min, three trains of high-frequency stimulation (HFS) were applied to induce long-term potentiation (LTP). Each train consisted of 100 pulses delivered at 100 Hz, with a 30-s interval between trains. The stimulation intensity remained constant throughout the LTP recording. The initial slopes of fEPSPs recorded prior to HFS were measured and used as the baseline, with subsequent responses expressed as a percentage of this baseline level. For paired-pulse ratio (PPR) recordings, a second stimulus was delivered following the first with intervals of 50 ms and 90 ms. The PPR was calculated as the ratio of the second fEPSP amplitude to the first. For pharmacological experiments, SAR405 (1 μM) or a vehicle was added to the perfusing ACSF to assess its effects on LTP and PPR.

### 2.10 Statistical analysis

All data in this study were analyzed using GraphPad Prism version 8.0 (GraphPad Software). All experiments were biologically replicated at least three times. Dots in the figure represented single mice or independent experiments. The sample sizes were described in the relevant figure legends. Comparisons between two groups were evaluated using an unpaired Student’s *t*-test. For multiple group comparisons, one-way analysis of variance (ANOVA) followed by Tukey’s test was applied. Fear conditioning training results were assessed using repeated two-way ANOVA with Bonferroni’s post-hoc test. Statistical tests were shown in each figure’s legend. Data were considered as statistically significant when *P*-values were <0.05 (*), <0.01(**), <0.001 (***) or <0.0001 (****). All data were shown as mean ± SEM.

## 3 Results

### 3.1 Fear conditioning increases Nrbf2 mRNA and protein levels

**FIGURE 1:**
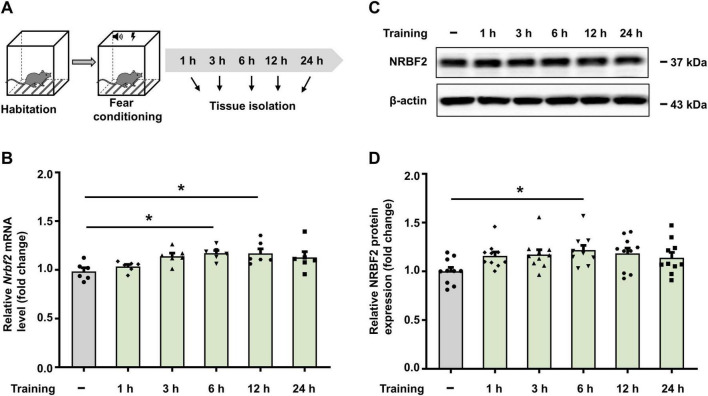
Fear conditioning increases *Nrbf2* mRNA and protein levels. **(A)** Schematic of experimental design for hippocampus tissue extraction. **(B)** Quantitative PCR results showing the *Nrbf2* mRNA level in the hippocampus at 1 h, 3 h, 6 h, 12 h and 24 h post-fear conditioning (*n* = 6 mice/group, one-way ANOVA with Tukey’s test, *F_5_,_30_* = 3.721, *p* = 0.009; *post hoc*.

To map the time window of *Nrbf2* mRNA and protein levels after fear conditioning, qPCR and WB were employed to assess NRBF2 levels in whole hippocampal tissue from wild-type mice at 1 h, 3 h, 6 h, 12 h and 24 h after fear conditioning, as well as the naïve group, which did not undergo fear conditioning (Figure 1A). The results showed that compared to the naïve group, the level of *Nrbf2* mRNA was significantly elevated at 6 h and 12 h after training (Figure 1B) (*F_5_,_30_* = 3.721, *p* = 0.009), while the NRBF2 protein level showed a significant increase at 6 h after training (Figures 1C, D) (*F_5_,_54_* = 2.451, *p* = 0.045), indicating the involvement of NRBF2 in fear memory formation.

### 3.2 Depletion of NRBF2 had no effect on locomotive activity and anxiety-like behavior

**FIGURE 2:**
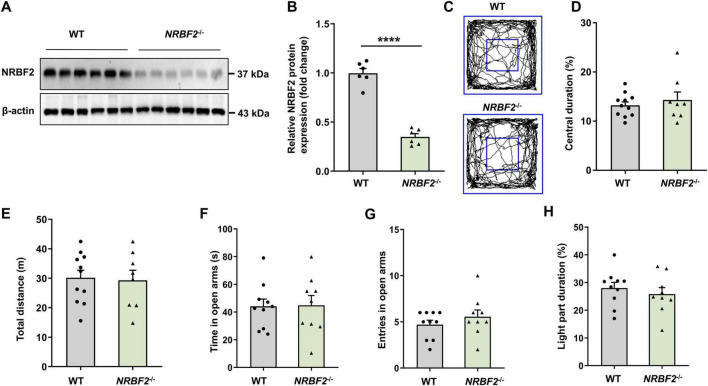
NRBF2-KO has no effect on locomotion and anxiety-like behavior. **(A,B)** Representative WB images **(A)** and quantification data **(B)** assess expression of NRBF2 in the hippocampus of mice (*n* = 6 mice/group, unpaired *t*-test, *t*_10_ = 11.07, ****p < 0.0001). **(C)** Representative traces showing the movement of NRBF2-KO and WT mice in the open field test. **(D,E)** Time spent in center zone **(D)** and total distance traveled **(E)** in the open field test (*n* = 11 mice in WT and 8 in NRBF2-KO group; **(D)** unpaired *t*-test, *t*_17_ = 0.685, *p* = 0.503; **(E)** unpaired *t*-test, *t*_17_ = 0.210, *p* = 0.836). **(F,G)** Time spent **(F)** and entries **(G)** in the open arms of the elevated plus maze test (*n* = 10 mice in WT and 9 in NRBF2-KO group; **(F)** unpaired *t*-test, *t*_17_ = 0.088, *p* = 0.931; **(G)** unpaired *t*-test, *t*_17_ = 1.005, *p* = 0.329). **(H)** The percentage of time spent in the light compartment of the light-dark box test (*n* = 10 mice in WT and 9 in NRBF2-KO group; unpaired *t*-test, *t*_17_ = 0.708, *p* = 0.488). Data were shown as mean ± SEM.

To substantiate the role of NRBF2 in learning and memory, we utilized NRBF2-KO mice (Yang et al., 2017), which were confirmed by complete elimination of NRBF2 protein in the hippocampus through WB analysis (Figures 2A, B) (*t*_10_ = 11.07, ****p < 0.0001). Subsequently, we investigated the potential influence of NRBF2 depletion on locomotor and anxiety-like behaviors by assessing behavioral changes in NRBF2-KO and wild-type (WT) mice using an open field test (OFT), elevated plus maze (EPM) test, and light-dark (LD) box test. Our results showed no significant alterations in exploratory behaviors, a key indicator of anxiety-like behaviors, as evidenced by the comparable the percentage of time spent in central area of the OFT (Figures 2C, D) (*t*_17_ = 0.685, *p* = 0.503), the time spent in the open arms (Figure 2F) (*t*_17_ = 0.088, *p* = 0.931) and entries into the open arms (Figure 2G) (*t*_17_ = 1.005, *p* = 0.329) of the EPM, as well as the percentage of time spent in the light compartment of the LD box (Figure 2H) (*t*_17_ = 0.708, *p* = 0.488) between NRBF2-KO and WT mice. Furthermore, the total distance traveled by the NRBF2-KO mice in the OFT was not significantly different from that of their control counterparts (Figure 2E) (*t*_17_ = 0.210, *p* = 0.836). These data show that NRBF2 depletion does not affect motor function and anxiety-like behavior.

### 3.3 Depletion of NRBF2, but not Vps34 inhibition, impaired memory acquisition

**FIGURE 3:**
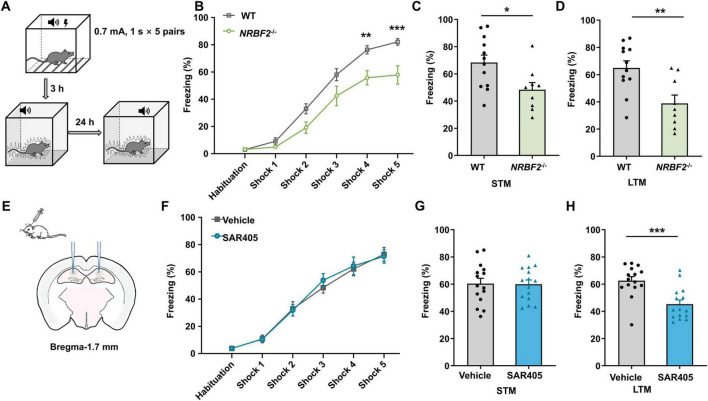
Effects of NRBF2 Deletion and SAR405 treatment on each of the fear memory stages. **(A)** Schematics of the procedure of fear conditioning. **(B)** The freezing curves during the training session showed the memory acquisition process in NRBF2-KO mice (*n* = 12 mice in WT and 9 in NRBF2-KO group, repeated two-way ANOVA, *F*_1,19_ = 11.05, *p* = 0.004; *post hoc*.

In light of the observed elevation in NRBF2 levels following fear conditioning, we conducted fear conditioning tests to assess the influence of NRBF2 depletion on fear memory, quantifying freezing behavior percentage (Figure 3A). Notably, during fear training, NRBF2-KO mice displayed a significantly lower freezing percentage compared to WT mice, indicating impaired learning (Figure 3B) (*F*_1,19_ = 11.54, *p* = 0.003). Furthermore, NRBF2-KO mice exhibited reduced freezing behavior at both short-term memory (STM, 3 h) (Figure 3C) (*t*_19_ = 2.550, *p = 0.02) and long-term memory (LTM, 24 h) intervals (Figure 3D) (*t*_19_ = 3.276, **p = 0.004), suggesting a deficit in memory acquisition affecting both STM and LTM (Bush et al., 2010; Makkar et al., 2010). Our findings underscored that NRBF2 deletion impaired memory acquisition, along with subsequent STM and LTM. Comparable performance in the OFT, EPM and LD tests excluded the possibility that alterations in freezing behaviors stemmed from variations in locomotor activity or anxiety-related behaviors.

It’s well-recognized that NRBF2 plays a pivotal role as a component of the Vps34 complex and regulates complex kinase activity in autophagy induction (Ohashi, 2021). Our previous study (Li et al., 2019) demonstrated that Vps34 inhibition by SAR405 in the amygdala impairs memory consolidation, so we further investigated the impact of Vps34 inhibition in the hippocampus on learning and memory. This investigation aimed to elucidate whether NRBF2 deletion yields similar effects to Vps34 inhibition. SAR405, a selective inhibitor of Vps34 commonly used for autophagy inhibition (Schlütermann et al., 2018; An and Harper, 2018; Ronan et al., 2014), was locally delivered to the bilateral hippocampus before fear conditioning (Figure 3E). Our findings revealed that SAR405 treatment impaired freezing behaviors at 24 h after training (*t*_28_ = 4.027, ***p < 0.001), with no differences observed during fear training (*F*_1,28_ = 0.044, *p* = 0.835) and in STM (*t*_28_ = 0.097, *p* = 0.932) (Figures 3F–H), Thus, our results suggested that SAR405 pretreatment specifically attenuated fear memory consolidation while leaving memory acquisition unaffected.

### 3.4 Inhibition of Vps34 with SAR405 does not impact excitatory synaptic transmission and long-term potentiation

**FIGURE 4:**
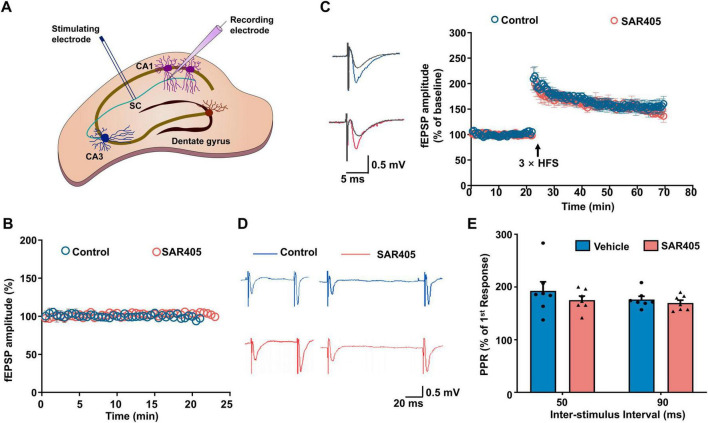
SAR405 does not affect synaptic plasticity in the hippocampus. **(A)** Schematic diagram of the field excitatory postsynaptic potential (fEPSP) in Schaffer collateral-CA1 pyramidal cells. **(B)** Baseline fEPSP slope was recorded after slices were treated with SAR405 or vehicle (n_vehicle_ = 4 slices, 4 mice, n_SAR405_ = 4 slices, 4 mice). **(C)** The baseline and LTP recording (n_vehicle_ = 6 slices, 5 mice, n_SAR405_ = 6 slices, 4 mice). **(D)** Representative traces of paired-pulse stimulation, which were evoked with inter-stimulus intervals of 50 ms and 90 ms in SAR405 treated and vehicle mice. **(E)** PPR was not significantly different in SAR405 treated compared with vehicle mice (n_vehicle_ = 7 slices, 4 mice, n_SAR405_ = 7 slices, 4 mice, repeat two-way ANOVA, *F*_1,6_ = 0.453, *p* = 0.526). Result is presented as mean ± SEM.

Enhanced synaptic plasticity in the hippocampus constitutes the fundamental cellular mechanism for spatial learning and memory, and limiting LTP expression has been shown to result in a deficit in fear acquisition, which subsequently translates into disrupted fear memory (Nabavi et al., 2014; Takeuchi et al., 2014). A recent study has reported a reduced maintenance of LTP in NRBF2-KO animals compared to WT (Lachance et al., 2019), consistent with our findings that NRBF2 deletion impaired memory acquisition. However, it remains unclear whether these deficits in memory acquisition and LTP are mediated through the Vps34 complex-induced autophagy mechanism. To address this question, we investigated the effects of SAR405 on synaptic transmission and LTP. Electrophysiological recordings were conducted to measure the field excitatory postsynaptic potential (fEPSP) in the CA3-CA1 region, and high-frequency stimulation (HFS) was employed to induce LTP. Our results, as depicted in Figures 4A–C, revealed that SAR405 treatment of the slices did not affect baseline fEPSP or HFS-induced LTP compared to the vehicle group. Furthermore, to investigate whether SAR405 affects presynaptic mechanisms, we measured the paired-pulse ratio (PPR), which is an indicator of presynaptic neurotransmitter release. We observed no significant change in the PPR ratio (Figures 4D, E) (*F*_1,6_ = 0.453, *p* = 0.526), indicating that SAR405 did not affect the probability of releasing vesicles at the presynaptic terminal. Taken together, these data suggest that the inhibition of Vps34 with SAR405 is not necessary for excitatory synaptic transmission and LTP.

## 4 Discussion

**FIGURE 5:**
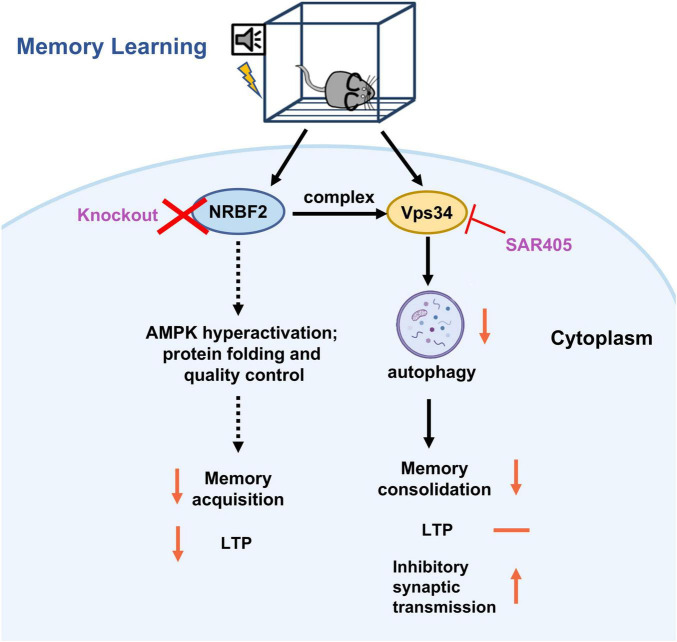
The schematic representation illustrates the effects and underlying mechanisms of NRBF2 on the memory acquisition process. During the learning and memory process, knockout of NRBF2, a component of the Vps34 complex, disrupts fear memory acquisition and LTP, potentially through the hyperactivation of AMPK (Lachance et al., 2019) and its involvement in protein folding and quality control (Ouyang et al., 2020). In contrast, inhibition of Vps34 by SAR405 selectively impairs memory consolidation by increasing inhibitory synaptic transmission (Li et al., 2019), without affecting memory acquisition and LTP.

The present study demonstrates that the levels of *Nrbf2* mRNA and protein increase following fear conditioning, and depletion of NRBF2 impairs fear memory acquisition as well as subsequent STM and LTM. Although NRBF2 is a key component of the Vps34 complex involved in autophagy induction, pharmacological inhibition of Vps34 by SAR405 selectively impairs memory consolidation without affecting memory acquisition or LTP (Figure 5). Our study provides evidence that NRBF2 influences memory acquisition in a non-autophagy dependent manner, while the induction of autophagy by the Vps34 complex is necessary for memory consolidation.

NRBF2 has been reported to be necessary for learning and memory (Ouyang et al., 2020), as well as cognitive impairment in Alzheimer’s disease (AD) patients (Yang et al., 2017; Lachance et al., 2019). Previous researches have demonstrated contextual memory deficits in NRBF2-KO animals through various cognitive tests, including radial-arm maze, fear conditioning, object location task and water maze (Lachance et al., 2019; Ouyang et al., 2020). Consistent with these findings, our study observed increased NRBF2 levels in the hippocampus following fear conditioning and demonstrated memory deficits upon NRBF2 deletion. Moreover, loss of NRBF2 does not affect motor abilities or anxiety-like behaviors in mice, but has been shown to induce a depression-like phenotype (Zhang et al., 2023). Additionally, we delineated the temporal dynamics of fear memory upon NRBF2 deletion and unequivocally showed that NRBF2 contributes to the acquisition stage of learning and memory.

NRBF2 has been identified as a protein that interacts with the Vps34 complex to induce autophagy (Behrends et al., 2010; Cao et al., 2014). Our previous research demonstrated that the infusion of the Vps34 inhibitor SAR405 into the amygdala disrupted memory consolidation by interfering with autophagy and inhibitory neurotransmission, while leaving memory acquisition unaffected (Li et al., 2019). Several other studies have also showed that autophagy inhibition, whether pharmacologically (3-MA and Spautin-1) or genetically (knockdown of *Atg7*, *BECN1*, or *LC3B*), impairs LTM while leaving STM intact (Pandey et al., 2021; Hylin et al., 2018). Consistent with these studies, our results demonstrated that SAR405 infusion into the hippocampus impaired memory consolidation. Collectively, these data confirm that inhibiting autophagy does not affect learning acquisition and STM.

In addition to the disparities observed in memory acquisition and consolidation between NRBF2 knockdown and autophagy inhibition, their impacts on synaptic plasticity also differ in cellular mechanisms. Autophagy has been shown to mediate the degradation of PSD-95 in synapses, which is required for NMDA receptor-dependent LTD (Compans et al., 2021). Pharmacological inhibition of autophagy using rapamycin and trehalose blocked LTD but had no effect on LTP and paired-pulse ratio (Kallergi et al., 2022). Similarly, genetic inhibition of autophagy through *Atg5* knockout and *Beclin-1* siRNAs showed that autophagy is necessary for LTD induction but not for LTP (Shen et al., 2020). Consistent with these findings, we found that SAR405 did not affect basal synaptic transmission and LTP in the hippocampus. However, deletion of NRBF2 led to impaired LTP maintenance (Lachance et al., 2019). These observations support the hypothesis that NRBF2 may regulate learning and memory through non-autophagy pathways (Ouyang et al., 2020; Ohashi, 2021).

Several potential mechanisms have been proposed to explain the regulation of memory acquisition by NRBF2. Hyperactivation of AMP-activated protein kinase (AMPK) and reduced activity of the mammalian target of rapamycin (mTOR) observed in NRBF2-KO mice may contribute to impaired memory and LTP (Lachance et al., 2019). Notably, AMPK has been shown to modulate LTP, with its activation partially inhibits LTP maintenance by suppressing the mTOR pathway (Herzig and Shaw, 2018; Kim et al., 2011). Moreover, mTOR is involved in the establishment of long-lasting LTP by mediating LTP-related protein synthesis (Tsokas et al., 2007). However, further direct evidence is needed to validate this hypothesis, particularly regarding the involvement of the AMPK-mTOR signaling pathway in autophagy regulation (Alers et al., 2012). Another inference from RNA-seq analysis suggested that NRBF2 regulated learning and memory by modulating networks associated with the retinoic acid receptors, protein folding, and quality control (Ouyang et al., 2020). Given that memory formation requires extensive mRNA transcription and protein translation, it is conceivable that NRBF2 may contribute to these processes. However, elucidating the specific mechanisms underlying these hypotheses will require further exploration through multi-omics and behavioral experiments.

## 5 Conclusion

Collectively, our study has provided convincing evidence that NRBF2 deletion impairs memory acquisition through an autophagy-independent pathway. These findings offer new insights into the role of NRBF2 and highlight the potential of targeting NRBF2 as a therapeutic strategy for addressing cognitive deficits associated with various disorders.

## Data Availability

The original contributions presented in this study are included in this article/supplementary material, further inquiries can be directed to the corresponding author.
